# A structural equation model study on the influencing factors of sleep disorders in patients with breast cancer undergoing chemotherapy

**DOI:** 10.3389/fonc.2025.1633763

**Published:** 2025-12-12

**Authors:** Ting Lu, Jiejing Wei, Rongsheng Xiong, Yi Xu

**Affiliations:** 1 Day Chemotherapy Center, The First Affiliated Hospital of Guangxi Medical University, Nanning, China; 2 Department of Oncology, The First Affiliated Hospital of Guangxi Medical University, Nanning, China; 3 Department of Thoracic Surgery, Nanxishan Hospital of Guangxi Zhuang Autonomous Region (The Second People's Hospital of Guangxi Zhuang Autonomous Region), Guilin, China

**Keywords:** breast cancer, chemotherapy, sleep disorders, psychological distress, pain, social support, coping strategies, structural equation modeling

## Abstract

**Objective:**

Sleep disorders are prevalent among breast cancer patients undergoing chemotherapy and are influenced by multiple psychological, social, and physiological factors. This study aims to explore the determinants of sleep disturbances in this population using a structural equation model (SEM), focusing on the role of social support, psychological distress, coping strategies, and pain.

**Methods:**

A cross-sectional study was conducted with 383 breast cancer patients undergoing chemotherapy from the Department of Oncology, the First Affiliated Hospital of Guangxi Medical University, Guangxi, China, from May 2023 to June 2024. The survey questionnaire contained a general data questionnaire and the Pittsburgh Sleep Quality Index (PSQI), Numeric Rating Scale (NRS), Simplified Coping Style Questionnaire (SCSQ), Social Support Rating Scale (SSRS), and Hospital Anxiety and Depression Scale (HADS). Structural equation modeling (SEM) was used to examine direct and indirect pathways affecting sleep quality. Model fit was assessed using CMIN/DF, RMSEA, GFI, AGFI, NFI, TLI, and CFI.

**Results:**

The SEM demonstrated good model fit (CMIN/DF = 2.061, RMSEA = 0.053, CFI = 0.959). Social support negatively correlated with psychological distress (β = -0.158, *P* = 0.013) and positively influenced sleep quality (β = -0.122, *P* = 0.028). Psychological distress (β = 0.567, *P* < 0.001) and pain (β = 0.191, *P* < 0.001) had significant negative effects on sleep quality. Mediation analysis confirmed that psychological distress significantly mediated the effects of social support and pain on sleep quality.

**Conclusions:**

Social support and psychological distress are key determinants of sleep quality in breast cancer patients undergoing chemotherapy. Psychological distress mediates the relationship between pain, social support, and sleep disturbances, emphasizing the need for psychosocial interventions to improve sleep quality in this population.

## Introduction

Breast cancer is the most prevalent malignant tumor among women worldwide (1), with chemotherapy serving as a cornerstone treatment to improve survival rates (2). However, chemotherapy is often accompanied by a range of adverse effects, including pain, fatigue, and psychological distress, all of which contribute to high rates of sleep disturbances in this population (3). Studies indicate that up to 65% of breast cancer patients undergoing chemotherapy experience significant sleep disturbances, including difficulty initiating sleep, frequent nighttime awakenings, and reduced sleep efficiency (4). Poor sleep quality not only exacerbates cancer-related symptoms but also increases treatment-related toxicity, compromises quality of life, and may even elevate the risk of recurrence or mortality (5). Despite the high prevalence of sleep disorders among breast cancer patients, the underlying mechanisms remain inadequately studied, particularly the complex interactions between psychological status, social support, pain, and coping strategies. A deeper understanding of these factors is crucial to developing targeted interventions aimed at improving sleep quality in this vulnerable population.

The biopsychosocial model provides a robust theoretical framework for understanding the multifaceted nature of sleep disturbances in breast cancer patients (6, 7). Within this model, psychological distress has been widely recognized as a key determinant of sleep disorders (8). Chronic stress and heightened nervous system arousal prolong sleep latency, increase nocturnal awakenings, and reduce deep sleep stages. Moreover, pain—a common symptom among chemotherapy patients—has been shown to disrupt sleep architecture, increasing wakefulness and reducing sleep quality (9). Beyond physiological factors, social support plays a crucial role in mitigating psychological distress and enhancing sleep quality (10). Studies suggest that strong social support networks are associated with lower anxiety and depression levels, which in turn improve sleep outcomes (11). Additionally, coping strategies significantly influence how patients manage psychological distress, further impacting sleep quality (12). Positive coping strategies, such as cognitive restructuring and problem-solving, have been linked to better psychological resilience and improved sleep, while maladaptive coping styles, such as avoidance and emotional suppression, may worsen sleep disturbances. However, the interactions among these variables in breast cancer patients remain unclear.

Given the complexity of these relationships, this study employs structural equation modeling (SEM) to simultaneously assess the direct and indirect pathways between social support, psychological status, coping strategies, pain, and sleep quality in breast cancer patients undergoing chemotherapy. SEM allows for a more comprehensive analysis of the interconnections between these factors, enabling the identification of key mediators and moderators. By constructing and validating an SEM-based model, this research aims to provide empirical evidence for targeted psychosocial and pain management interventions that can enhance sleep quality and overall well-being in breast cancer patients. The findings of this study will contribute to the development of evidence-based interventions to improve the quality of life for this high-risk population.

## Methods

### Formulation of research hypotheses

#### Theoretical framework and research hypotheses

This study is based on the Biopsychosocial Model as the theoretical foundation for understanding the influencing factors of sleep disorders in breast cancer patients undergoing chemotherapy. The model integrates biological (pain), psychological (anxiety and depression), behavioral (coping strategies), and social (social support) factors to provide a comprehensive explanation of sleep disturbances in this population. Sleep disorders are often associated with complex interactions between these factors, making a multidimensional approach essential.

Based on this framework, the study formulated the following hypotheses (**Table 1**).

**Table 1:** Research hypotheses.

Hypothetical	Research hypothesis
H1	Social support negatively correlates with psychological distress (HADS), implying that higher social support reduces anxiety and depression.
H2	Psychological distress negatively affects sleep quality (PSQI), where increased anxiety and depression lead to poorer sleep.
H3	Social support indirectly improves sleep quality through its impact on psychological distress.
H4	Pain exerts a direct effect on sleep quality while also influencing it indirectly via psychological distress.
H5	Coping strategies mediate the relationship between social support and psychological distress, influencing sleep quality.
H6	Coping strategies have a direct effect on sleep quality (PSQI), where active coping improves sleep, and passive coping worsens sleep outcomes.

**Figure 1:**
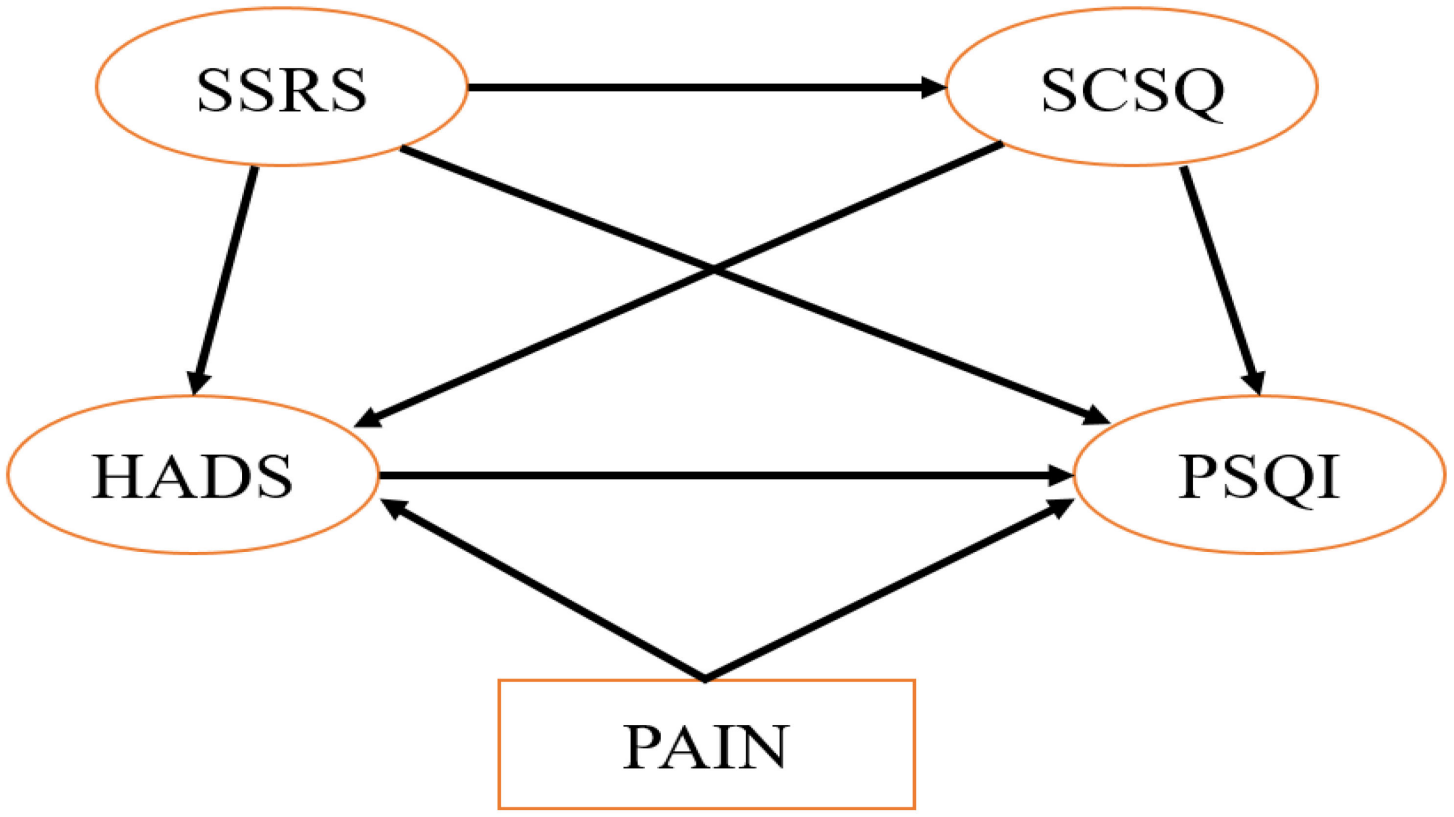
Analysis model of sleep disorders in patients with breast cancer undergoing chemotherapy.

To test these hypotheses, SEM was employed, which allows the simultaneous analysis of multiple interrelated pathways. Finally, a sleep disorder analysis model for breast cancer patients undergoing chemotherapy was constructed (**Figure 1**).

### Sample selection

A cross-sectional study was conducted at the Department of Oncology, the First Affiliated Hospital of Guangxi Medical University, Guangxi, China, from May 2023 to June 2024. Sample inclusion criteria: (1) patients who undergoing chemotherapy and had been diagnosed breast cancer; (2) age ≥ 18; (3) No severe cardiovascular, hepatic, renal dysfunction, or other major diseases; (4) No severe psychiatric disorders or cognitive impairments, capable of understanding and following intervention requirements. (5) patients who gave informed consent and voluntarily participated in the study. Exclusion criteria: (1) patients with severe audio-visual impairment and an inability to communicate; (2) patients in poor condition who had difficulties completing the survey questionnaire.

### Sample size calculation

The sample size required for this study was determined using an online Sample Size Calculator for SEM, available at https://wnarifin.ocpu.io/sscalc/www/ssrmsea.html. The calculation was based on the Root Mean Squared Error of Approximation (RMSEA) method, which is widely used for estimating sample size requirements in SEM studies (13). The parameters were set for the calculation: Expected RMSEA = 0.05, df=77, α=0.05, 1-β=90%, Expected dropout rate=20%. Based on these inputs, the minimum required sample size was calculated to be 231 participants. Considering a 20% dropout rate, the final recommended sample size for recruitment was 289 participants. However, to enhance the robustness and reliability of the study findings, a total of 383 patients were ultimately included in the final analysis, exceeding the estimated requirement to account for potential missing data and ensure adequate statistical power.

### Ethical statement

This study was approved by the Ethics Committee of the First Affiliated Hospital of Guangxi Medical University (Approval No. Z-A20230453). Written informed consent was obtained from all participants prior to data collection, and all procedures were conducted in accordance with the Declaration of Helsinki.

### Survey instruments

In order to ensure the scientific validity of the study and the good reliability of the questionnaire, all the measurement items in this study were referred to existing, well-established scales and adapted to the context of this study. The survey covered five areas: sleep quality, social support, psychological status, coping style, and pain.

### Sleep quality

The Pittsburgh Sleep Quality Index (PSQI), developed by Buysse et al. in 1989 (14). It is a self-rated questionnaire which assesses sleep quality and disturbances over 1 month time interval. Nineteen individual items generate seven “component” scores: subjective sleep quality, sleep latency, sleep duration, habitual sleep efficiency, sleep disturbances, use of sleeping medication, and daytime dysfunction. The sum of scores for these seven components yields one global score. Higher scores indicate worse sleep quality. A global PSQI score greater than 5 yielded a diagnostic sensitivity of 89.6% and specificity of 86.5% in distinguishing good and poor sleepers. The Cronbach’s alpha was 0.84.

### Psychological status

The Hospital Anxiety and Depression Scale (HADS), Developed by Zigmond and Snaith, 1983 (15). It is a self-rated instrument for anxiety and depression symptoms in the past week. It has seven items for anxiety and seven items for depression (14 total items, with each score ranging from 0 to 3) and has been widely used for people with cancer (16). The scores for these seven items are then added for a single total score with a range of 0–21 for HADS anxiety and HADS depression. HADS has demonstrated good reliability with a Cronbach’s alpha ranging from 0.68 to 0.93 (17).

### Social support

The Social Support Rating Scale (SSRS), developed by Xiao Shuiyuan according to China’s national conditions with good reliability and validity (18). It includes 10 items in three dimensions: subjective support, objective support and support availability. The sum of the scores of 10 items is the total score. Higher scores indicate stronger support networks.

### Coping style

The Simplified Coping Style Questionnaire (SCSQ), developed by Xie (19). It is a 20-question self-report scale divided into two dimensions: positive coping style and negative coping style. It is used to assess a person’s coping style when dealing with things. These questions are rated on a scale from 1 (never) to 4 (always). The total score of SCSQ is the positive dimension standard score minus the negative dimension standard score. The higher the score, the more likely the individual is to use positive coping styles. The Cronbach’s alpha was 0.9.

### Pain

The Numerical Rating Scale (NRS) (20), is an 11-point scale comprising a number from 0 through 10; 0 indicates “no pain”, and 10 indicates the “worst imaginable pain”, with higher scores indicating more severe pain. Patients are instructed to choose a single number from the scale that best indicates their level of pain.

### Data acquisition

This study applied an electronic questionnaire and paper questionnaires to complete data collection by a person on a one-to-one basis. Prior to the start of the survey, all questionnaire entries and survey technical details were discussed in detail by the four investigators to ensure consistency in the research process. Accompanied by the medical and nursing staff of the department, a professionally trained investigator introduces the purpose and content of the survey to the patients, seeks their informed consent, and then invites them to participate in this survey. The survey was limited to 10–20 min per respondent.

### Data collection

A questionnaire survey was conducted on patients who were able to cooperate with the study, and information was collected and evaluated when the patients were admitted to the hospital for chemotherapy. The collected information included the patients’ general information, disease-related information, and the patients’ sleep conditions, coping styles, social support, and anxiety and depression were assessed.

### Quality control

Investigators received training to ensure data consistency. The training content included research objectives, questionnaire filling requirements, and evaluation methods for each scale. Trained investigators stated point by point and completed the questionnaire based on the patient’s answers. The questionnaires were distributed and collected on site to check for omissions, verification, and recall. The data were recorded by two researchers, and questionnaires with errors or omissions exceeding 20% ​​or completely similar were eliminated.

### Statistical methods

All data were entered using Microsoft Excel and analyzed using SPSS 27.0 and AMOS 25.0 software. Descriptive statistics were used to summarize participants’ sociodemographic and clinical characteristics. Categorical variables were expressed as frequencies and percentages N (%), while continuous variables were presented as means with standard deviations (Mean ± SD). Independent samples t-tests and one-way ANOVA were conducted to compare sleep quality scores across different groups. Pearson correlation analysis was used to explore bivariate relationships between key variables, including sleep quality, psychological distress, social support, pain, and coping strategies. P-value of <0.05 was considered statistically significant. SEM was employed to test the hypothesized relationships among social support, coping strategies, psychological distress, pain, and sleep quality. Model fit was assessed using multiple goodness-of-fit indices following established SEM guidelines. The chi-square to degrees of freedom ratio (CMIN/DF) was used as an overall measure of model fit, with values < 3.0 indicating acceptable fit. The root mean square error of approximation (RMSEA) evaluates approximate fit; values ≤ 0.08 indicate acceptable fit, and values ≤ 0.05 indicate good fit. The goodness-of-fit index (GFI) and adjusted goodness-of-fit index (AGFI) assess absolute model fit, with recommended thresholds of ≥ 0.90. Incremental fit was evaluated using the normed fit index (NFI), Tucker–Lewis index (TLI), and comparative fit index (CFI), for which values ≥ 0.90 indicate acceptable fit and values ≥ 0.95 indicate excellent fit. These criteria align with widely accepted recommendations for SEM model evaluation (21, 22). The model was iteratively modified based on modification indices and theoretical considerations to achieve the best fit. Mediation effects were tested using bias-corrected bootstrapping with 2000 samples to estimate indirect effects and 95% confidence intervals. P-value < 0.05 was deemed statistically significant.

## Results

### Baseline demographic and clinical characteristics

A total of 383 breast cancer patients undergoing chemotherapy were enrolled in this study. The majority were middle-aged (48.3%) and married (86.7%), with most diagnosed at stage II of breast cancer (60.1%). Radical surgery was the most frequently adopted treatment (72.8%). In terms of ethnicity, Han and Zhuang populations predominated. Nearly half of the participants (48.6%) had two children, and a significant proportion resided in urban areas (42.3%). Regarding education and income, 37.3% of participants held a university degree or higher, while 40.7% reported a monthly income below CNY 3, 000. Physical activity levels were generally low, with 62.7% engaging in minimal physical activity and only 0.5% reporting high levels of exercise. These findings reflect considerable sociodemographic and clinical diversity across the sample, which provides a comprehensive foundation for subsequent structural equation modeling analysis (**Table 2**).

**Table 2:** Baseline demographic and clinical data of patients.

Category	Subcategory	Frequency	Percentage (%)
Age	Youth	158	41.3
	Middle-aged	185	48.3
	Elderly	40	10.4
Cancer Stage	I	52	13.6
	II	230	60.1
	III	78	20.4
	IV	23	6
Surgical Method	No surgery	29	7.6
	Breast-conserving surgery	75	19.6
	Radical surgery	279	72.8
Ethnicity	Zhuang	161	42
	Han	206	53.8
	Others	16	4.2
Marital Status	Married	332	86.7
	Others	51	13.3
Occupation	Farmers/Workers	132	34.5
	Employees/Self-employed	126	32.9
	Retired/Others	125	32.6
Education Level	Primary school or below	39	10.2
	Junior high school/Vocational school	130	33.9
	High school	71	18.5
	University or above	143	37.3
Residence	Urban	162	42.3
	Town	109	28.5
	Rural	112	29.2
Number of Children	≥3 children	50	13.1
	2 children	129	33.7
	1 daughter/1 son	186	48.6
	No children	18	4.7
Monthly Income (CNY)	<3000	156	40.7
	3000~5000	153	39.9
	>5000	74	19.3
Physical Activity Level	Sedentary	53	13.8
	Low activity	240	62.7
	Moderate activity	88	23
	High activity	2	0.5

### Sleep quality scores of breast cancer patients undergoing chemotherapy

The overall PSQI score among breast cancer patients undergoing chemotherapy was 8.20 ± 4.12, indicating generally poor sleep quality. Among the subcomponents, sleep onset time had the highest mean score, reflecting difficulties in falling asleep, followed by reduced sleep efficiency. In contrast, the use of sleep medication had the lowest mean score. These data present a detailed profile of sleep disturbances in this population (**Table 3**).

**Table 3:** Sleep quality scores of breast cancer patients undergoing chemotherapy.

Item	Score (Mean ± SD)
Subjective Sleep Quality	1.36 ± 0.83
Sleep Onset Time	2.52 ± 1.78
Sleep Duration	1.30 ± 0.94
Sleep Efficiency	2.20 ± 1.32
Sleep Disturbance	0.71 ± 0.65
Sleep Medication Use	0.11 ± 0.43
Daytime Dysfunction	1.50 ± 0.96
PSQI	8.20 ± 4.12

### Comparison of sleep conditions in breast cancer patients undergoing chemotherapy with different characteristics

An analysis of sleep scores among breast cancer patients undergoing chemotherapy revealed no statistically significant differences across demographic and clinical variables (P > 0.05). Sleep quality did not significantly vary by age, cancer stage, surgical method, ethnicity, marital status, occupation, education level, residence, number of children, income level, or physical activity level. While some subgroups, such as retired individuals or those without children, had numerically higher PSQI scores, these differences did not reach statistical significance (**Table 4**).

**Table 4:** Comparison of sleep score in breast cancer patients undergoing chemotherapy with different characteristics.

Item	Category	N (%)	Score (Mean ± SD)	t/F	*P*
Age	Youth	158 (41.3)	7.77 ± 3.94	1.619	0.199
	Middle-aged	185 (48.3)	8.57 ± 4.34		
	Elderly	40 (10.4)	8.18 ± 3.68		
Tumor Stage	I	52 (13.6)	7.83 ± 4.27	0.22	0.883
	II	230 (60.1)	8.32 ± 3.91		
	III	78 (20.4)	8.10 ± 4.57		
	IV	23 (6.0)	8.13 ± 4.44		
Surgical Method	No surgery	29 (7.6)	8.59 ± 4.10	1.119	0.328
	Breast-conserving surgery	75 (19.6)	7.57 ± 3.89		
	Radical surgery	279 (72.8)	8.32 ± 4.18		
Ethnicity	Zhuang	161 (42.0)	7.98 ± 4.03	1.047	0.352
	Han	206 (53.8)	8.26 ± 4.22		
	Others	16 (4.2)	9.50 ± 3.67		
Marital Status	Married	332 (86.7)	8.06 ± 4.17	-1.790	0.078
	Others	51 (13.3)	9.08 ± 3.72		
Occupation	Farmers/Workers	132 (34.5)	7.80 ± 4.49	1.716	0.181
	Employees/Self-employed	126 (32.9)	8.09 ± 3.99		
	Retired/Others	125 (32.6)	8.73 ± 3.81		
Education Level	Primary school or below	39 (10.2)	4.68 ± 0.75	0.833	0.476
	Junior high school/Vocational school	130 (33.9)	4.02 ± 0.35		
	High school	71 (18.5)	3.77 ± 0.45		
	University or above	143 (37.3)	4.22 ± 0.35		
Residence	Urban	162 (42.3)	8.42 ± 4.02	0.472	0.624
	Town	109 (28.5)	7.94 ± 3.92		
	Rural	112 (29.2)	8.13 ± 4.45		
Number of Children	≥3 children	50 (13.1)	8.78 ± 4.43	0.472	0.624
	2 children	129 (33.7)	7.60 ± 4.33		
	1 daughter/1 son	186 (48.6)	8.39 ± 3.81		
	No children	18 (4.7)	8.78 ± 4.57		
Monthly Income (CNY)	<3000	156 (40.7)	8.46 ± 4.19	0.698	0.498
	3000~5000	153 (39.9)	7.91 ± 4.17		
	>5000	74 (19.3)	8.23 ± 3.87		
Physical Activity Level	Sedentary	53 (13.8)	4.00 ± 0.55	1.130	0.337
	Low activity	240 (62.7)	4.30 ± 0.28		
	Moderate activity	88 (23.0)	3.67 ± 0.39		
	High activity	2 (0.5)	3.54 ± 2.50		

### Correlation analysis

As shown in **Table 5**, sleep quality was significantly correlated with several key psychological and physical factors. PSQI scores were positively associated with psychological distress (*r* = 0.451, *P* < 0.01) and pain levels (*r* = 0.261, *P* < 0.01), suggesting that greater anxiety, depression, and pain were linked to poorer sleep quality. In contrast, social support (SSRS) demonstrated a negative correlation with PSQI (*r* = –0.184, *P* < 0.01), indicating a protective role of support networks in sleep. Additionally, HADS was significantly related to both pain (*r* = 0.182, *P* < 0.01) and SSRS (*r* = –0.150, *P* < 0.01), further reinforcing the interconnectedness of psychological and social variables. Notably, coping strategies (SCSQ) were not significantly correlated with sleep quality (*r* = 0.038, *P* > 0.05).

**Table 5:** Correlation analysis.

Item	PSQI	SCSQ	SSRS	HADS	Pain
PSQI	1	0.038	-0.184^**^	.451^**^	0.261^**^
SCSQ	0.038	1	0.373^**^	-0.041	0.037
SSRS	-0.184^**^	.373^**^	1	-.150^**^	-0.009
HADS	0.451^**^	-0.041	-0.150^**^	1	0.182^**^
Pain	0.261^**^	0.037	-0.009	.182^**^	1

PSQI, Pittsburgh Sleep Quality Index; SCSQ, Simplified Coping Style Questionnaire; SSRS, Social Support Rating Scale; HADS, Hospital Anxiety and Depression Scale; Pain: Self-reported pain level. **P<0.01.

### Model construction of influencing factors of sleep disorders in breast cancer chemotherapy patients

**Figure 2:**
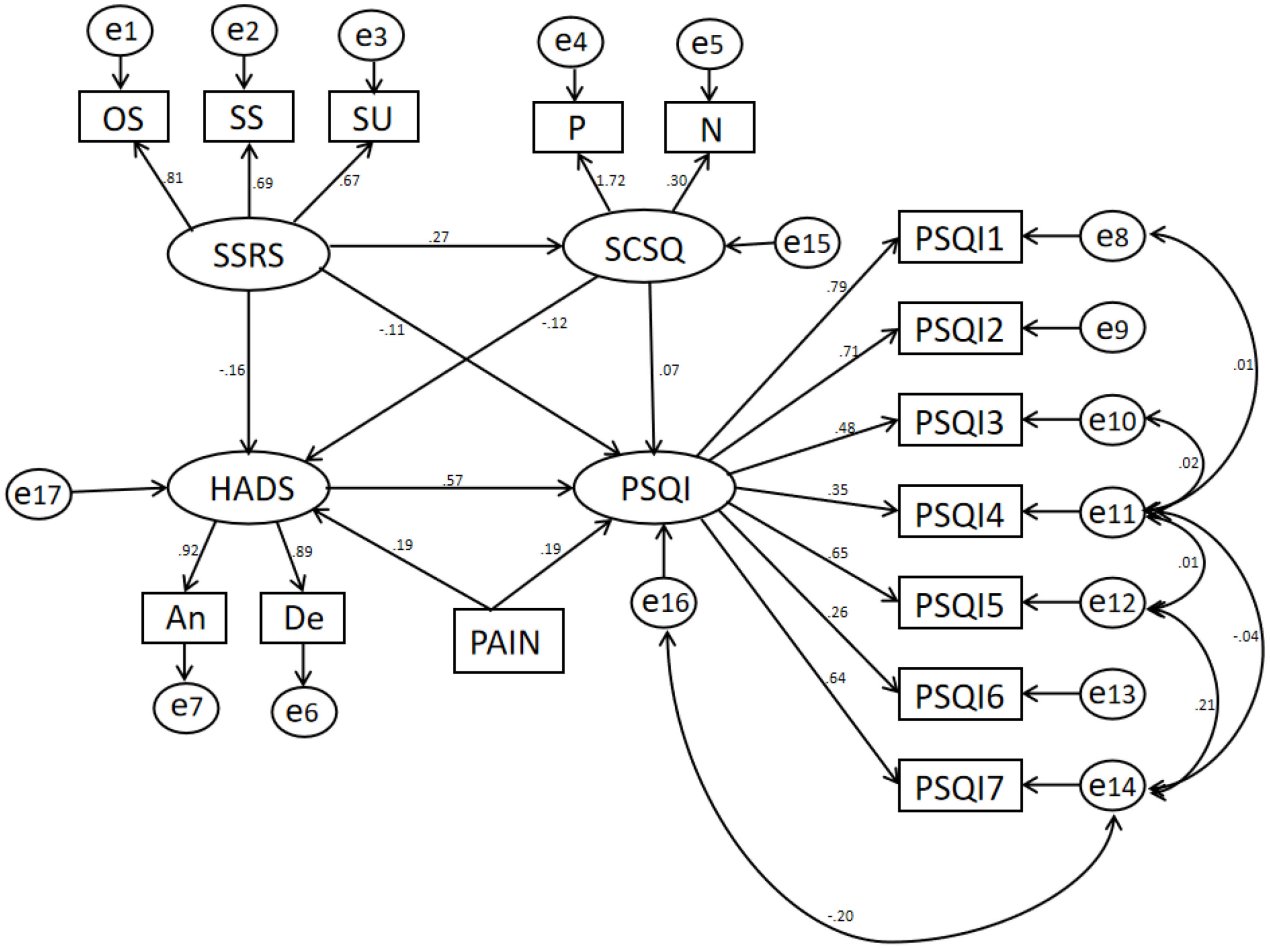
Model of factors influencing sleep disorders in breast cancer chemotherapy patients.

Note: The SEM analyzing the factors influencing sleep quality (PSQI) in breast cancer patients undergoing chemotherapy. The model includes several latent variables: social support (SSRS), coping style (SCSQ), hospital anxiety and depression (HADS), and pain, with their corresponding observed indicators. SSRS (Social Support) is influenced by three observed variables: OS (Objective Support), SS (Subjective Support), and SU (Support Utilization).SCSQ (coping style) is influenced by positive (P) and negative (N) components. HADS (Hospital Anxiety and Depression Scale) is determined by Anxiety (An) and Depression (De). The measurement model includes PSQI subcomponents (PSQI1 to PSQI7) as observed variables linked to PSQI, illustrating how sleep quality is assessed.

The SEM analysis revealed the interrelationships among social support, psychological status, coping strategies, pain, and sleep quality in breast cancer patients undergoing chemotherapy (**Figure 2**). Social support (SSRS) was negatively associated with psychological distress (HADS), indicating that higher levels of social support correlated with lower anxiety and depression. Additionally, psychological distress exhibited a significant positive association with sleep disturbances, suggesting that increased anxiety and depression negatively impacted sleep quality. Pain was found to exert both direct and indirect effects on sleep quality (PSQI). The direct pathway indicated that higher pain levels were associated with poorer sleep quality, while the indirect effect, mediated through psychological distress, reinforced this relationship. Coping strategies (SCSQ) were incorporated into the model to assess their moderating role. However, the influence of coping strategies on sleep quality did not reach statistical significance, suggesting that coping mechanisms might not independently affect sleep outcomes in this patient population. These findings underscore the critical role of psychological distress and pain in determining sleep quality among breast cancer patients undergoing chemotherapy.

### Model fit results

The structural equation model demonstrated acceptable fit. The CMIN/DF was 2.061, within the recommended threshold of <3. The RMSEA was 0.053, indicating a good fit (<0.08). Fit indices including GFI (0.949), AGFI (0.921), NFI (0.924), TLI (0.944), and CFI (0.959) all exceeded the standard cutoff of 0.90, indicating satisfactory model performance (**Table 6**).

**Table 6:** Model fit results.

Fit Index	CMIN/DF	RMSEA	GFI	AGFI	NFI	TLI	CFI
Fit Criteria	<3	<0.08	>0.90	>0.90	>0.90	>0.90	>0.90
Computed Results	2.061	0.053	0.949	0.921	0.924	0.944	0.959

### Results of path analysis

The structural equation modeling analysis provided empirical support for several of the proposed hypotheses (**Table 7**). H1 was supported, as social support (SSRS) was negatively associated with psychological distress (HADS) (β = -0.158, P = 0.013), indicating that patients with higher social support experienced lower levels of anxiety and depression. H2 was also confirmed, with psychological distress showing a strong positive association with sleep disturbance (PSQI) (β = 0.567, P < 0.001), suggesting that increased psychological distress contributed to poorer sleep quality. H3, proposing that social support indirectly improves sleep quality through its effect on psychological distress, was partially supported: social support showed a direct negative effect on PSQI (β = -0.122, P = 0.028) and an indirect pathway via HADS. H4 was validated, as pain had both a direct positive effect on sleep disturbance (β = 0.191, P < 0.001) and an indirect effect mediated by its significant positive influence on psychological distress (β = 0.192, P < 0.001). In contrast, H5, which hypothesized that coping strategies (SCSQ) mediate the relationship between social support and psychological distress, was not supported. Although social support positively predicted coping strategies (β = 0.266, P < 0.001), coping strategies did not significantly affect psychological distress (β = -0.111, P = 0.145). Similarly, H6 was not supported, as coping strategies showed no significant direct effect on sleep quality (β = 0.067, P = 0.176).

**Table 7:** Results of path analysis.

Dependent variable	Direction	Independent variable	Unstandardized path coefficient	Standardized path coefficient	S.E.	C.R.	*P*
HADS	<—	SSRS	-0.149	-0.158	0.060	-2.472	0.013
PSQI	<—	HADS	0.099	0.567	0.010	9.686	***
PSQI	<—	SSRS	-0.020	-0.122	0.009	-2.197	0.028
PSQI	<—	Pain	0.064	0.191	0.016	4.061	***
HADS	<—	Pain	0.371	0.192	0.099	3.749	***
SCSQ	<—	SSRS	0.942	0.266	0.118	8.018	***
HADS	<—	SCSQ	-0.030	-0.111	0.020	-1.458	0.145
PSQI	<—	SCSQ	0.003	0.067	0.002	1.353	0.176

***P<0.001.

These findings indicate that while social support and pain influence sleep quality both directly and indirectly via psychological distress, coping strategies did not have a statistically significant role in the pathway model.

### Mediation effect test

The mediation analysis further confirmed the hypothesized indirect effects among the key variables. In support of H3, psychological distress (HADS) significantly mediated the relationship between social support (SSRS) and sleep quality (PSQI). The total indirect effect was 0.089 (P < 0.005), while the direct effect remained significant at 0.122, resulting in a total effect of 0.211. This indicates that social support improves sleep quality both directly and indirectly by reducing psychological distress. Similarly, H4 was supported by the finding that psychological distress also mediated the association between pain and sleep quality. The total mediation effect of pain through HADS was 0.109 (P < 0.005), while the direct effect of pain on PSQI was 0.191, yielding a total effect of 0.300. These findings confirm that pain contributes to sleep disturbances not only through a direct pathway but also by exacerbating psychological distress, which in turn impairs sleep quality. Overall, the mediation analysis highlights the central role of psychological distress as a mediator linking both social and biological factors to sleep outcomes in this population.

## Discussion

This study investigated the factors influencing sleep disturbances in breast cancer patients undergoing chemotherapy using a SEM approach. The results revealed that psychological distress (HADS) and pain levels (NRS) were significant predictors of sleep quality (PSQI), with higher psychological distress and pain leading to poorer sleep outcomes. Social support (SSRS) was found to have an indirect effect on sleep quality, primarily mediated through its impact on psychological distress. Coping strategies (SCSQ) did not show a statistically significant direct effect on sleep quality. The path analysis confirmed that pain significantly influenced both psychological distress and sleep quality, suggesting that pain management is crucial for improving sleep among these patients. The model fit indices indicated a good fit, supporting the robustness of the proposed framework. These findings highlight the importance of psychological well-being, pain management, and social support interventions in mitigating sleep disturbances in breast cancer patients undergoing chemotherapy.

To further contextualize these findings, several psychophysiological mechanisms may help explain how pain and psychological distress contribute to sleep disturbance in this population. Cognitive–emotional hyperarousal, maladaptive sleep-related beliefs, and attentional bias toward somatic sensations contribute to a heightened state of arousal that interferes with normal sleep regulation (23). Patients experiencing pain or elevated psychological distress may become overly attentive to bodily cues and develop negative interpretations of sleep difficulties, which further exacerbate sleep disruption (24). These mechanisms provide theoretical support for the mediating role of psychological distress found in our structural equation model.

Building on these mechanisms, anxiety and depression—two key components of psychological distress—may influence sleep through distinct pathways. Anxiety is more strongly associated with difficulties in initiating sleep, whereas depression tends to contribute to early morning awakenings and reduced total sleep time. Using a combined HADS score may therefore obscure these differential effects, and future studies may consider analyzing anxiety and depression separately to better elucidate their specific contributions to sleep disturbance.

Another plausible explanation for the non-significant association between coping strategies and sleep quality is that coping was modeled as a single composite construct in this study. Coping is inherently multidimensional, and different subtypes—such as avoidant versus approach-based coping, and emotion-focused versus problem-focused coping—may have divergent effects on psychological distress and sleep (25, 26). Some maladaptive strategies, such as avoidance, may exacerbate distress without directly influencing sleep, whereas more adaptive, problem-focused coping may reduce stress but still fail to produce immediate improvements in sleep outcomes. In addition, cultural characteristics common among Chinese cancer patients, including collectivistic values, family-centered decision-making, and cancer-related stigma, may shape coping preferences and lead individuals to rely more on internalized or avoidant strategies (27). These culturally influenced tendencies may partly explain why coping did not demonstrate a direct effect on sleep in our model. Future research should distinguish coping subtypes to clarify their specific roles in the psychosocial pathways affecting sleep.

The findings of this study align with previous research on the association between psychological distress, pain, and sleep disturbances in cancer patients. Prior studies have demonstrated that higher levels of anxiety and depression significantly impact sleep quality, leading to increased sleep latency, poorer sleep efficiency, and greater daytime dysfunction (28, 29). Similarly, our study confirmed that psychological distress, as measured by the HADS, had a significant negative effect on sleep quality, consistent with these earlier reports. Furthermore, pain has been well-documented as a major contributor to sleep disturbances in cancer patients, with studies indicating that pain interferes with sleep continuity and exacerbates nocturnal awakenings (30). Our findings reinforced this relationship, showing that pain directly impacted both psychological distress and sleep quality, emphasizing the necessity of pain management interventions in improving sleep outcomes.

However, a key difference between our study and previous research lies in the role of coping strategies. While some studies have suggested that adaptive coping mechanisms significantly improve sleep quality in cancer patients (31), our results did not identify a statistically significant direct effect of coping style on sleep quality. This discrepancy may be due to differences in measurement tools, cultural factors, or variations in coping strategy effectiveness depending on patient characteristics. Additionally, while social support has been widely recognized as a protective factor in mental health and sleep quality (32, 33), our findings indicate that its effect on sleep was mediated through psychological distress rather than having a direct impact, which has been similarly observed in other cancer populations (34). These findings contribute to a more nuanced understanding of how psychological, behavioral, and physiological factors interact in influencing sleep disturbances in breast cancer patients undergoing chemotherapy.

This study employed SEM to analyze the complex relationships between social support, psychological distress, pain, coping strategies, and sleep quality among breast cancer patients undergoing chemotherapy. Unlike conventional regression-based analyses, SEM allows for simultaneous examination of direct and indirect pathways between multiple variables, providing a more comprehensive understanding of causal mechanisms (35). The model demonstrated a good fit with the data, suggesting that the hypothesized relationships were well-supported. This advanced statistical method has been increasingly utilized in psycho-oncology research (36, 37).

One novel aspect of our study is the identification of psychological distress as a key mediator in the relationship between social support and sleep quality. While previous studies have established that social support improves sleep quality (38), our results suggest that its effect is indirect and mediated through reductions in anxiety and depression. This finding highlights the need for psychosocial interventions that not only enhance social support but also target mental health to achieve improvements in sleep outcomes. Another unique contribution is the examination of pain as both a direct and indirect determinant of sleep disturbances. Our results indicate that pain directly impairs sleep quality, while also increasing psychological distress, further exacerbating sleep problems. These findings underscore the multifaceted nature of sleep disturbances in breast cancer patients and emphasize the importance of comprehensive symptom management strategies that integrate pain control, psychological support, and sleep interventions. Furthermore, while previous research has suggested a significant role of coping strategies in mental health and sleep quality (39), our study did not find a statistically significant direct effect of coping strategies on sleep. This result suggests that coping strategies may function more as an intermediary factor influencing psychological distress rather than directly affecting sleep quality. Future research should further explore the role of different coping styles in relation to long-term sleep outcomes and whether specific coping interventions could enhance psychological resilience and improve sleep patterns in breast cancer patients.

## Conclusion

This study highlights the complex interplay of social support, psychological distress, pain, and coping strategies in influencing sleep disturbances among breast cancer patients undergoing chemotherapy. By employing a structural equation model, the findings emphasize the need for a multidimensional approach to improving sleep quality in this population. Psychological distress and pain were identified as key contributors to poor sleep, suggesting that targeted interventions focusing on mental health support and pain management may be beneficial. Moreover, the role of social support in alleviating sleep disturbances underscores the importance of strengthening support networks for patients during treatment. While this study provides valuable insights, future research should consider longitudinal designs to establish causality and explore individualized interventions incorporating cognitive-behavioral and psychosocial strategies. Additionally, integrating objective sleep measures alongside self-reported data could enhance the accuracy of sleep assessments. Addressing these factors through a multidisciplinary approach may improve not only sleep outcomes but also the overall well-being of breast cancer patients during chemotherapy.

## Limitations and future directions

Despite its strengths, this study has several limitations. First, the cross-sectional design limits the ability to establish causal relationships between psychological distress, coping strategies, and sleep disturbances. Second, the reliance on self-reported measures introduces potential recall bias and social desirability bias, which may affect the accuracy of responses. Third, the study was conducted in a single tertiary hospital, which may limit the generalizability of the findings to broader populations. Future research should employ longitudinal designs to establish causality, incorporate objective sleep measures such as actigraphy, and expand sample diversity across multiple centers to enhance generalizability. Additionally, exploring biological markers and developing targeted interventions, may provide further insights into improving sleep quality in breast cancer patients undergoing chemotherapy.

## Data Availability

The raw data supporting the conclusions of this article are available from the corresponding author on reasonable request.
