# Correction: A structural equation model study on the influencing factors of sleep disorders in patients with breast cancer undergoing chemotherapy

**DOI:** 10.3389/fonc.2026.1768585

**Published:** 2026-01-13

**Authors:** Ting Lu, Jiejing Wei, Rongsheng Xiong, Yi Xu

**Affiliations:** 1 Day Chemotherapy Center, The First Affiliated Hospital of Guangxi Medical University, Nanning, China; 2 Department of Oncology, The First Affiliated Hospital of Guangxi Medical University, Nanning, China; 3 Department of Thoracic Surgery, Nanxishan Hospital of Guangxi Zhuang Autonomous Region (The Second People’s Hospital of Guangxi Zhuang Autonomous Region), Guilin, China

**Keywords:** breast cancer, chemotherapy, sleep disorders, psychological distress, pain, social support, coping strategies, structural equation modeling

Affiliation 1 and Affiliation 2 were erroneously presented in the incorrect order.

The correct affiliations are as follows:

1. Day Chemotherapy Center, The First Affiliated Hospital of Guangxi Medical University, Nanning, China

2. Department of Oncology, The First Affiliated Hospital of Guangxi Medical University, Nanning, China

The original version of this article has been updated.

